# Untargeted mass spectrometry discloses plasma solute levels poorly controlled by hemodialysis

**DOI:** 10.1371/journal.pone.0188315

**Published:** 2017-11-16

**Authors:** Tammy L. Sirich, Pavel A. Aronov, Jonathan Fullman, Khanh Nguyen, Natalie S. Plummer, Timothy W. Meyer

**Affiliations:** The Departments of Medicine, VA Palo Alto HCS and Stanford University, Palo Alto, CA, United States of America; University Medical Center Utrecht, NETHERLANDS

## Abstract

Many solutes have been reported to remain at higher plasma levels relative to normal than the standard index solute urea in hemodialysis patients. Untargeted mass spectrometry was employed to compare solute levels in plasma and plasma ultrafiltrate of hemodialysis patients and normal subjects. Quantitative assays were employed to check the accuracy of untargeted results for selected solutes and additional measurements were made in dialysate and urine to estimate solute clearances and production. Comparison of peak areas indicated that many solutes accumulated to high levels in hemodialysis patients, with average peak areas in plasma ultrafiltrate of dialysis patients being more than 100 times greater than those in normals for 123 features. Most of these mass spectrometric features were identified only by their mass values. Untargeted analysis correctly ranked the accumulation of 5 solutes which were quantitatively assayed but tended to overestimate its extent. Mathematical modeling showed that the elevation of plasma levels for these solutes could be accounted for by a low dialytic to native kidney clearance ratio and a high dialytic clearance relative to the volume of the accessible compartment. Numerous solutes accumulate to high levels in hemodialysis patients because dialysis does not replicate the clearance provided by the native kidney. Many of these solutes remain to be chemically identified and their pathogenic potential elucidated.

## Introduction

In patients treated by conventional hemodialysis, many solutes remain at much higher plasma levels relative to normal than the index solute urea. The degree to which solute levels are elevated, however, varies widely [1,2]. We have previously reported that the degree to which solute levels are elevated depends heavily on two factors [3]. One factor is the ratio of the dialytic clearance to the native kidney clearance. When the dialytic clearance is low relative to the native kidney clearance, plasma levels in dialysis patients tend to remain high. The other factor is the ratio of the dialytic clearance to the volume of distribution. When the dialytic clearance is high relative to the accessible volume of distribution, not much more solute is removed toward the end of each treatment. This makes intermittent treatment inefficient. The reduction ratio with treatment is high, but peak and time-averaged plasma solute levels remain elevated.

Determination of plasma solute levels in dialysis patients and normal subjects requires quantitative chemical assays. Most reports assessing the degree of solute accumulation in patients have thus examined only a limited number of compounds. Untargeted mass spectrometry can be employed to estimate the relative levels of numerous compounds in dialysis patients and controls without determining absolute molar concentrations [4,5,6,7]. Untargeted mass spectrometry can also distinguish the accumulation of solutes which have not yet been chemically identified [8]. The current study employed high resolution untargeted mass spectrometry to identify additional solutes which are poorly cleared by hemodialysis and therefore rise to prominent levels in the plasma of patients. For selected solutes, results obtained by untargeted mass spectrometry were compared with those obtained by quantitative assays.

## Materials and methods

### Untargeted mass spectrometry

Measurements were made in samples from 6 patients without residual function on hemodialysis and 6 subjects with normal kidney function which were collected as previously reported [3,9]. Normal subjects provided timed urine samples and plasma samples in the morning after an overnight fast. Characteristics of the subjects are summarized in S1 Table. Patients provided pre- and post-treatment plasma and dialysate samples and subjects with normal kidney function provided plasma and urine samples. The study was approved by the Stanford institutional review board and participants gave informed consent. Studies were performed in accordance with the Declaration of Helsinki. Further details of sample processing including preparation of ultrafiltrate for estimation of free solute concentrations are provided in the S1 Methods.

Untargeted analysis was performed using liquid chromatography (LC) coupled with high resolution mass spectrometry (MS). LC was performed on an ACQUITY UPLC system (Waters, Milford, MA). Samples were loaded to a Kinetex XB-C18 150 x 2.1 mm, 1.7 μm particle size column (Phenomenex). Mobile phase flow was 0.4 ml/min using 0.1% formic acid in water (A) and 0.1% formic acid in acetonitrile (B) with gradient from 3% B to 25% B over 9 minutes, from 25% B to 100% B to 15 minutes, remaining at 100% B to 19 minutes and returning to 3% B to 21 minutes as previously described [9]. MS analysis was performed on a QExactive mass spectrometer (ThermoFisher, San Jose, CA) with data collected over the range of m/z 70 to 800 using electrospray ionization (ESI) with heated probe and positive/negative ion switching. Each sample was run in triplicate.

Sieve software v2.1 (ThermoFisher, San Jose, CA) was used to identify features characterized by retention time and mass to charge ratio (m/z) and to assign amplitudes based on peak area integration of the ion current values. Adducts and isotopes were removed by the software and features considered not to represent defined chromatographic peaks were removed by visual inspection. Analysis of the mass spectrograms was performed in two steps. Features present in the pre-treatment plasma and plasma ultrafiltrate from the 6 hemodialysis patients were first identified. These features were then searched for in the entire sample set.

A feature was considered present in a sample if peaks with assignable areas were detected in at least 2 of the 3 replicates, and the average peak area was then calculated. Using this approach, 741 features were detected in at least 5 of 6 samples of pre-treatment plasma and/or plasma ultrafiltrate from hemodialysis patients (559 features in the negative mode and 182 features in the positive mode). Removal of duplicates detected in both modes left 706 features for further analysis as listed in S2 Table.

Efforts to identify features identified by untargeted mass spectrometry were focused on those with high peak areas in plasma ultrafiltrate from dialysis patients compared to normal subjects. Compounds with matching mass were sought for in the Human Metabolome Database [10]. Chemical reagent standards were acquired as listed in S3 Table for 63 compounds with mass values corresponding to 49 features with peaks areas in patients' plasma ultrafiltrate averaging 174 times greater than in normal subjects' plasma ultrafiltrate. Solute identity was confirmed by matching the retention times and mass fragmentation patterns of features in subjects to those of the reagent standards using the same LC method and an Orbitrap Fusion mass spectrometer (ThermoFisher, San Jose, CA). Chemical identity of 14 of the 706 features observed in dialysis patients' plasma and/or plasma ultrafiltrate was confirmed by this analysis.

### Quantitative mass spectrometry

A new LC/MS/MS assay was developed to provide quantitative measurement of homovanillic acid sulfate (HVAS) which was identified as accumulating to high levels by untargeted analysis. Samples were prepared as for untargeted analysis with further processing of urine samples by solid phase extraction (SPE) using Oasis WAX 30 mg sorbent cartridges (Waters, Milford, MA) as described in the S1 Methods. For LC/MS/MS, 10 μl of each sample was loaded on a Synergi Hydro-RP 30x2 mm, 2.5 mcm particle size column (Phenomenex, Torrance, CA) maintained at 30°C. Buffer flow was 0.4 ml/min using 0.1% formic acid in water (A) and 0.1% formic acid in acetonitrile (B) with 0% B for 1 min, then 0% B to 80% B over 2 min, 80% B over 0.4 min, 80% to 0% B over 0.1 min and 0% B for 2.5 min. MS was performed on an Agilent 6430 Triple Quadrupole with ESI in the negative mode (Agilent Technologies, Santa Clara, CA). Homovanillic acid sulfate-d3 was used as the isotopic internal standard (HVAS-d3, Santa Cruz Biotechnology, Santa Cruz, CA). Ion transitions for quantitation were m/z 261 → 181 for HVAS and 264 → 184 for HVAS-d3. Recoveries are summarized in S1 Methods. Previously described assays were used to measure phenylacetylglutamate, hippuric acid, indoxyl sulfate, and p-cresol sulfate [11].

### Other assays, calculations and statistics

Untargeted data was analyzed using mass spectrometric peak areas as surrogates for solute concentrations. Dialytic clearances were estimated as the amount removed in the dialysate divided by the logarithmic mean of the pre- and post-treatment plasma concentrations and daily solute production was estimated as half the amount removed at the midweek dialysis session. Native kidney clearances were estimated from the urinary solute excretion rates and the plasma solute concentrations in normal subjects, and solute production rates were assumed equal to the urinary excretion rates. The degree of solute accumulation in dialysis patients was estimated as the ratio of the average peak area in pre-treatment samples to the average peak area in normal samples, and values were calculated only when peak areas were measurable in at least two normal samples as well as in at least five pre-treatment dialysis samples. A previously described mathematical model was employed to assess the extent to which the degree of solute accumulation in dialysis patients could be accounted for by the ratio of dialytic clearance to native kidney clearance and the fractional reduction in solute concentration during dialysis treatment [3]. Modeling was performed using Matlab (version 2014b, MathWorks, Natick, MA). To assess the significance of concentration differences in patients and normal subjects, concentration values were first log-transformed and then compared using the unpaired t-test. False discovery rates (q values) were then calculated from p values using Q-VALUE software (http://genomics.princeton.edu/storeylab/qvalue/). Features were characterized as uremic if they were detected in only one or in none of the normal samples or when the ratio of the average peak areas in the pretreatment samples compared to the normal samples (HD/NL) was greater than 4 and statistical analysis assigned a q value of < 0.05 to the difference. The HD/NL ratio of 4 for classification of solutes as uremic was arbitrarily set equal to the observed HD/NL plasma concentration ratio for urea. This assessment was made separately for plasma and plasma ultrafiltrate. Values obtained by quantitative mass spectrometry in dialysis patients and normal subjects were compared using the unpaired t-test. The fasting and postprandial values in normal subjects were compared using the paired t-test. Urea was measured in the plasma and urine by the clinical laboratory and in dialysate by UV photospectrometry.

## Results

A Total of 706 features considered to represent individual solutes were detected in the pretreatment samples of plasma ultrafiltrate and/or plasma in at least five of six maintenance dialysis patients. Their accurate mass values and relative peak areas are listed in S2 Table. A majority, including 573 of the total of 706, were characterized as uremic by comparison of peak areas in patients and controls. Using the criteria described in the methods, 465 were characterized as uremic based on peak areas in both plasma ultrafiltrate and plasma while 35 features were characterized as uremic by peak areas in plasma ultrafiltrate only and 73 were characterized as uremic by peaks areas in plasma only. Comparison of peak areas indicated further that many solutes accumulated to high levels in hemodialysis patients, with average peak areas in plasma ultrafiltrate of dialysis patients being more than 100 times greater than those in controls for 123 of the features characterized as uremic.

Most of the features characterized as uremic were identified only by their mass values. In the majority of cases, known biologic compounds with mass within 5 parts per million (ppm) were not found in the Human Metabolome Database [10], as further summarized in S2 Table. Fourteen compounds were identified by matching values for retention time, mass, and mass fragmentation with chemical standards as listed in Table 1. The average difference between mass assigned by the software and the known monoisotopic mass for these compounds was only 0.2±1.2 parts per million. For an additional 33 features identified as uremic in the current study, observed mass values matched those of known uremic solutes as listed in S4 Table. Reagent standards for these solutes were not obtained, however, so the identities of the features were not established.

**Table 1:** Kinetic behavior of chemically identified solutes.

Solute	[HD]/[NL] observed	K_HD_ (ml/min)	[UF]/[P]_HD_	Reduction Ratio (%)	K_NL_ (ml/min)	[UF]/[P]_NL_	K_HD_ / K_NL_	G_HD_ / G_NL_
pyrrolidonecarboxylic acid	373	166 ±7	1.08 ±0.17	82 ±5	633 ±347	1.08 ±0.26	0.26	4.0
N-2-furoyl glycine	352	136 ±47	0.68 ±0.20	68 ±11	760 ±599	0.60 ±0.17	0.18	3.1
alanyl-glycine	291	184 ±10	0.94 ±0.12	79 ±5	705 ±342	1.25 ±0.47	0.26	3.4
phenylacetylglutamine	285	170 ±7	1.02 ±0.16	81 ±5	517 ±167	0.91 ±0.17	0.33	3.5
4-pyridoxic acid	242	76 ±10	0.11 ±0.04	54 ±11	348 ±107	0.06 ±0.02	0.22	3.2
homovanillic acid sulfate	242	136 ±12	0.66 ±0.14	80 ±5	311±202	0.24 ±0.18	0.44	4.4
hippuric acid	58	143 ±14	0.55 ±0.07	70 ±5	595 ±211	0.32 ±0.07	0.24	0.6
isovaleryglycine	32	201 ±18	1.03 ±0.08	70 ±5	270 ±143	1.03 ±0.26	0.74	0.9
indoxyl sulfate	30	52 ±11	0.10 ±0.03	40 ±11	89 ±34	0.03 ±0.01	0.58	1.1
cinnamoylglycine	27	37 ±16	0.11 ±0.04	22 ±28	224 ±114	0.01 ±0.01	0.17	0.4
p-cresol sulfate	15	52 ±8	0.09 ±0.02	37 ±10	37 ±8	0.03 ±0.01	1.41	1.2
salicylate	9	94 ±33	0.19 ±0.06	44 ±14	13 ±9	0.10 ±0.03	7.23	12.2
indoxyl glucuronide		168 ±11	0.82 ±0.06	75 ±6				5.9
phenylglucuronide		188 ±16	1.02 ±0.12	80 ±6				4.0
urea	4	312 ±42	-	75 ±5	80 ±29	-	3.92	0.6

Values for non-urea solutes are estimated from peak areas obtained by untargeted mass spectrometry in plasma, dialysate, and urine. Values for urea obtained by standard chemical methods on the same samples are provided for comparison. [HD]/[NL], ratio of plasma solute concentration in predialysis compared to normal samples; K_HD_, dialytic clearance; [UF]/[P]_HD_, free solute fraction in predialysis plasma; K_NL_, native kidney clearance, [UF]/[P]_NL_, free solute fraction in normal plasma; K_HD_ / K_NL_, ratio of dialytic clearance to native kidney clearance; Reduction Ratio, concentration reduction during dialysis treatment. G_HD_ / G_NL_, rate of solute removal in patients' dialysate compared to normal subjects’ urine. [HD]/[NL], K_NL_, and [UF]/[P]_NL_ values were not calculated for indoxyl glucuronide and phenyl glucuronide because solute concentrations could be estimated in only 1 normal subject. K_NL_ could be estimated for isovaleryglycine in 5, for N-2-furoyl glycine in 4, and for homovanillic acid sulfate in 3 out of the 6 normal subjects. Urea was not measured in the ultrafiltrate so its [UF]/[P] was not calculated.

The estimated predialysis to normal concentration ratios for all of the 14 chemically identified uremic solutes exceeded the concentration ratio of 4 obtained for urea, and were in many cases much higher as summarized in Table 1. We sought to identify the kinetic properties responsible for the high solute concentrations in dialysis patients as shown in the table. The estimated dialytic clearances for the 14 solutes ranged from 37±16 to 201±18 ml/min. These dialytic clearance values were below urea's dialytic clearance of 312±42 ml/min. The lowest values were observed for solutes which were protein-bound as reflected by comparison of peak areas in plasma ultrafiltrate and plasma. The reduction in concentrations during treatment ranged from 22±28 to 82±5%, with the lower values observed for bound solutes with low dialytic clearances. In contrast to the dialytic clearances, native kidney clearances were higher than urea's native kidney clearance of 80±29 ml/min for all but 2 of the 12 solutes for which values could be calculated. As a result, for all of these solutes except salicylate the dialytic to normal clearance ratio was much lower than the value of 4 obtained for urea.

Kinetic parameters obtained by untargeted mass spectrometry were compared with values obtained by quantitative LC/MS/MS assays for five solutes, as summarized in Table 2. The degree of solute accumulation in predialysis compared to normal plasma was correctly ordered by the untargeted analysis. Its magnitude, however, was overestimated particularly for the solutes with the highest apparent concentration ratios. Clearance values were again correctly ordered but the untargeted analysis overestimated both the dialytic and native kidney clearances of homovanillic acid sulfate, indoxyl sulfate, and p-cresol sulfate. Values for the reduction ratio obtained with the untargeted and quantitative assays were more nearly similar and significantly different only for homovanillic acid sulfate.

**Table 2:** Results of quantitative compared to untargeted mass spectrometric analysis.

Solute	Assay	[HD]/[NL] observed	[HD]/[NL] modeled	K_HD_ (ml/min)	K_NL_ (ml/min)	K_HD_ / K_NL_	Reduction Ratio (%)	G_HD_ / G_NL_
phenylacetylglutamine	LC/MS/MS	126	65	181 ±12	404 ±95	0.45	81 ±5	2.2
	UN	285	95	170 ±7*	517 ±167*	0.33	81 ±5	3.5
homovanillic acid sulfate	LC/MS/MS	80	84	56 ±23	160 ±45	0.37	69 ±9	1.0
	UN	242	70	136 ±12*	311 ±202	0.44	80 ±5*	4.3
hippuric acid	LC/MS/MS	42	96	129 ±9	520 ±140	0.25	71 ±6	0.5
	UN	58	108	143 ±14	595 ±211	0.24	70 ±5	0.6
indoxyl sulfate	LC/MS/MS	28	33	34 ±6	65 ±22	0.52	40 ±12	1.0
	UN	30	31	52 ±11*	89 ±34*	0.58	40 ±11	1.1
p-cresol sulfate	LC/MS/MS	20	16	24 ±5	23 ±7	1.04	35 ±12	1.3
	UN	15	13	52 ±8*	37 ±8*	1.41	37 ±10	1.3
urea	-	4	7	312 ±42	80 ±29	3.92	75 ±5	0.60

Comparison of values for non-urea solutes assayed quantitatively by liquid chromatography/mass spectrometry with isotopic dilution (LC/MS/MS) and by untargeted mass spectrometry (UN); [HD]/[NL] observed, ratio of average plasma solute concentration in pre-dialysis compared to normal plasma; [HD]/[NL] modeled, ratio of plasma solute concentrations by mathematical modeling; K_HD_, dialytic clearance; K_NL_, normal kidney clearance; Reduction Ratio, fractional reduction in plasma levels during dialysis treatment; G_HD_ / G_NL_, rate of solute removal in patients' dialysate compared to normal subjects’ urine.

*, p <0.01 for LC/MS/MS value vs. UN value by t-test without adjustment for multiple comparisons. Values for urea were obtained on the same samples by standard chemical methods.

Observed predialysis to normal concentration ratios were compared to modeled values as further summarized in Table 2. The observed solute accumulation was greater than predicted for phenylacetylglutamine and less than predicted for hippuric acid. These differences could be accounted for by the differences in solute production rates which were revealed by comparison of solute removal in patients' dialysate and normal subjects' urine. A discrepancy in the results obtained with untargeted and quantitative results for homovanillic acid sulfate could be accounted for, however, only by failure of peak areas in the untargeted assay to accurately represent solute concentrations.

## Discussion

The number of known uremic solutes is increasing [2,7,12,13]. A major impetus to this growth has been the application of mass spectrometry. Uremic solutes were first identified by organic chemists in normal urine and then shown to accumulate in the plasma when the kidneys failed. Solutes present in high concentrations were therefore identified first, and the number detected has increased as analytic methods have improved. Mass spectrometry coupled to liquid chromatography affords increased sensitivity and has therefore led to identification of many additional uremic solutes [4,5,6,7,12].

Mass spectrometry affords not only increased sensitivity but the ability to detect large numbers of solutes in individual samples. The term "untargeted" is applied when mass spectrometry is employed to interrogate samples for the presence of numerous solutes without predetermination of those of interest. Untargeted analysis can be carried out in two ways [4,7,14]. First, mass spectrograms of samples can be compared with those of a panel of reagent standards. This method allows chemical identification of numerous solutes but will miss large numbers of compounds that are not included in the standard panel [14]. Alternatively, as in the current study, mass spectrograms can be analyzed to provide lists of features which represent compounds. This method can detect additional compounds but does not reveal their chemical identity.

Results of the current study suggest that many uremic solutes remain to be chemically identified. The number of mass spectrometric features characterized as uremic in the current study exceeds the number of known uremic solutes compiled from reviews of the literature [2,12,13]. For most of these 573 features, compounds with mass values within 5 ppm were not found in a standard database. An immediate question is whether the features identified by high-resolution untargeted mass spectrometry in fact represent biochemicals present in plasma. Various adducts and fragments of biochemicals can be generated when samples are analyzed by LC/MS. Analytic software is designed to sort out such features but cannot do so with complete accuracy. The list of features characterized as uremic in S2 Table thus undoubtedly includes some artifacts. The number of as yet unidentified compounds present in human plasma and other biologic fluids is thought, however, to be large [14,15]. Unidentified compounds that accumulate in uremia likely include conjugates of compounds derived from plant foods and from colon microbial metabolism. To establish the chemical identity of a mass spectrometric feature, however, requires comparison with the mass spectrograms of a chemical standard. In the current study, analysis of 68 standards established the chemical identity of only 14 mass spectrometric features as listed in Table 1. All of these solutes except alanyl-glycine had previously been identified as uremic.

Results of the current study suggest not only that there are a large number of unidentified uremic solutes but that many of their plasma levels remain very high in patients maintained on hemodialysis. As we have previously described, a low dialytic to native kidney clearance ratio combined with a large fractional reduction in the plasma concentration during treatment can account for prominent solute accumulation in dialysis patients [11]. For all of the uremic solutes that we chemically identified except salicylate, the estimated ratios of the dialytic clearance to native kidney clearance were lower than the average value of 4 observed for urea. These lower ratios reflect the failure of hemodialysis to replicate the secretory function of the native kidney and the uniquely high dialytic clearance of urea, magnified in some cases by restriction of the solute's dialytic clearances due to plasma protein-binding. Salicylate is a notable exception, in that it has a low native kidney clearance due to tubular reabsorption [16,17]. The current study was too small to distinguish the effect of dialyzer size on solute clearances. Previous work suggests, however, that increasing dialyzer size would only slightly increase the clearance of bound solutes, while a combined increase in dialyzer size and dialysate flow can significantly increase the clearance of protein-bound solutes [18,19,20]. New medium cut-off dialysis membranes would not be expected to increase the clearance of solutes in the mass range of 70 to 800 Da studied here but have been shown to increase the clearance of larger solutes [21,22].

The magnitude of solute accumulation in dialysis patients may also be influenced by differences in solute production. Dialysis patients in the current study produced more phenylacetylglutamine and less hippuric acid than normal subjects. Solute production rates may be affected by factors including diet, age, gender, and conditions such as diabetes. Prior studies using quantitative assays suggest that the influence of these factors can be detected only by studying very large numbers of subjects [23]. In normal subjects, diurnal variation in production may cause diurnal variation in solute levels as recently described by Rivara et al. [24].

This study also reveals the limitations in the accuracy with which untargeted mass spectrometry can determine solute concentrations. First, it should be emphasized that untargeted mass spectrometry assesses only relative solute concentrations in different samples and not molar concentrations. Second, other compounds present in samples can interfere variably with the ionization and delivery of each solute into the mass spectrometer [25]. These limitations were apparent in the assessment of homovanillic acid sulfate in the current study. The untargeted analysis suggested that homovanillic acid sulfate levels were over 200-fold normal in dialysis patients and that dialysis patients produced much more homovanillic acid sulfate than normal subjects. A quantitative LC/MS/MS assay with an isotopic internal standard assay was developed to check these apparent findings. Quantitative measurements revealed a hemodialysis to normal concentration ratio of about 80 without a notable difference in solute production. Untargeted mass spectrometry thus correctly identified homovanillic acid sulfate among solutes whose levels are poorly controlled by conventional hemodialysis, but overestimated the extent of its accumulation. We hope that future studies employing untargeted mass spectrometry will identify solutes associated with clinical outcomes in ESRD patients. Considerable effort can then be expended to determine the chemical identity of solutes for which untargeted mass spectrometry provides only accurate mass values.[26] And quantitative assays will be required to confirm the observed associations of solute levels with outcomes and to test the biologic activity of potentially toxic solutes.

Limitations of the current study should be emphasized. As described above, we may have identified as uremic of mass spectrometric features that were analytic artifacts and do not represent biochemicals present in dialysis patients' plasma. A probably greater limitation is failure to detect many true uremic solutes. We employed reverse phase chromtography and a mobile phase with an increasing concentration of the organic solvent acetonitrile. This method is not suitable for detection of strongly hydrophillic uremic solutes including many guanidines. We also employed a single ionization method. Greater numbers of solute can be detected when different analytic methods are combined. Studies combining a variety of methods have identified more than 4,000 named solutes in normal human plasma along with a greater number of features representing unknown compounds [14,27,28,29,30]. Analysis of a small sample set likely further limited our ability to categorize solutes as uremic.

Overall, the current study extends the literature suggesting that the number of uremic solutes is large, and further suggests that many solutes which accumulate to relatively high levels remain to be chemically identified. The problem of determining which uremic solutes are toxic thus appears increasingly complex.

## Supporting information

S1 Methods Sample processing for untargeted mass spectrometry analysis and for quantitative measurement of homovanillic acid sulfate by LC/MS/MS with isotopic dilution. (PDF)

S1 Table Characteristics of hemodialysis patients and normal subjects. (PDF)

S2 Table Features in Pre-Dialysis plasma ultrafiltrate and/or plasma detected by untargeted high resolution mass spectrometry. (PDF)

S3 Table Reagent compounds compared to uremic features. (PDF)

S4 Table Uremic features with mass values matching those of known uremic solutes for which reagent standards were not obtained. (PDF)
