# Supplementary material for: Untargeted mass spectrometry discloses plasma solute levels poorly controlled by hemodialysis
Source: PLoS One. 2017 Nov 16;12(11):e0188315. doi: 10.1371/journal.pone.0188315 (PMC5690664; doi:10.1371/journal.pone.0188315)
Supplement: S1 Methods — (PDF) [file pone.0188315.s001.pdf]

S1 Methods

Sample processing for untargeted mass spectrometry

Plasma was deproteinized with methanol (1:3 vol:vol), dried, and reconstituted in 95:5 water:acetonitrile to the original concentration for normal subjects and to one-fourth (pre-treatment) and one-half (post-treatment) the original concentration for hemodialysis patients. Ultrafiltrate was obtained using Nanosep 30K Omega separators, dried, and reconstituted in 95:5 water/acetonitrile to five times the original concentration for normal subjects and to the original (pre-treatment) and two times (post-treatment) the original concentration for hemodialysis patients. Urine was diluted to provide solute concentrations that would be found in a urine flow of 100 ml/min. Dialysate was dried and reconstituted in 95:5 water/acetonitrile to the original concentration.

Sample processing and recoveries for quantitative measurement of HVAS by LC/MS/MS with isotopic dilution

Plasma was deproteinized with methanol (1:3 vol:vol), dried, and reconstituted in 0.1% formic acid in water to the original concentration for normal subjects and to one-fourth (pre-treatment) and original (post-treatment) concentration for hemodialysis patients. Ultrafiltrate was obtained using Nanosep 30K Omega separators, dried, and reconstituted in 0.1% formic acid in water to five times the original concentration for normal subjects and run as original concentrations (pre-treatment and post-treatment) for hemodialysis patients. Dialysate was run as the original concentration. Urine was diluted with water to provide solute concentrations that would be found in a urine flow of 10 ml/min (U10). The U10 samples were further processed by solid phase extraction (SPE) using Oasis WAX 30 mg sorbent 1-ml syringe barrel cartridges (Waters, Milford, MA). All solvents were eluted through the SPE cartridge by applying manual pressure. The SPE cartridge was first equilibrated with 500 μl of water followed by 500 μl of

MeOH. Then 200 μl of U10 combined with 50 μl of homovanillic acid sulfate-d3 as an internal standard (HVAS-d3, Santa Cruz Biotechnology) were loaded onto the cartridge. The cartridge was washed with 250 μl of 2% formic acid in water and then with 250 μl of 100% MeOH before elution with 250 μl of 5% NH_4OH in MeOH. The eluent was dried and reconstituted in 0.1% formic acid in water.

Recoveries for HVAS were 106±20% for reagent added to plasma, 99±13% for reagent added to plasma ultrafiltrate, 104±7 for reagent added to urine, and 106±6 for reagent added to dialysate to achieve concentrations similar to those found in experimental subjects.
