# Supplementary material for: Untargeted mass spectrometry discloses plasma solute levels poorly controlled by hemodialysis
Source: PLoS One. 2017 Nov 16;12(11):e0188315. doi: 10.1371/journal.pone.0188315 (PMC5690664; doi:10.1371/journal.pone.0188315)
Supplement: S1 Table — (PDF) [file pone.0188315.s002.pdf]

S1 Table. Characteristics of Hemodialysis Patients and Normal Subjects

	Hemodialysis Patients (n=6)	Normal Subjects (n=6)
Age (yrs)	66 ± 14	43 ± 11
Gender (f/m)	0 / 6	2 / 4
Diabetes (yes/no)	3 / 3	0 / 6
Dialysis Vintage (yrs)	3 ± 2	
Treatment Durations (hrs)	3.3 ± 0.5	
Blood Flow Rate (ml/min)	392 ± 20	
Dialysate Flow Rate (ml/min)	795 ± 8	
Monthly $spKt/V_{urea}$	1.68 ± 0.30	

Values are mean ± sd. Dialyzers used in the hemodialysis patients were Revaclear (n=3) and Revaclear Max (n=3).
