# Supplementary material for: Untargeted mass spectrometry discloses plasma solute levels poorly controlled by hemodialysis
Source: PLoS One. 2017 Nov 16;12(11):e0188315. doi: 10.1371/journal.pone.0188315 (PMC5690664; doi:10.1371/journal.pone.0188315)
Supplement: S2 Table — (PDF) [file pone.0188315.s003.pdf]

S2 Table. Features in Pre-Dialysis Plasma Ultrafiltrate and/or Plasma Detected by Untargeted High Resolution Mass Spectrometry

Listed are all the 706 features detected in at least 5 of 6 samples of pre-treatment plasma ultrafiltrate and/or plasma from the patients on maintenance hemodialysis. Features are characterized by their exact mass in amu and the polarity of the ion source (NEG, negative; POS, positive) which allowed their detection. 35 features were detected in both positive and negative mode and for these features the polarity which provided the highest peak amplitudes is listed. A total of 573 features were characterized as uremic based on the appearance of higher concentrations in the predialysis samples than in normal samples of plasma ultrafiltrate and/or plasma. Concentrations in the plasma ultrafiltrate (UF) and/or plasma were considered to be higher than normal when the features was detected in only one or in none of the normal samples (indicated by *) or when the ratio of the average peak areas (HD/NL) in the predialysis samples compared to the normal samples was greater than 4 and statistical analysis assigned a q value of < 0.05 to this difference. At total of 465 features were characterized as uremic by these criteria based on findings in both plasma ultrafiltrate and plasma while 35 features were characterized as uremic by findings in plasma ultrafiltrate only and 73 were characterized as uremic by findings in plasma only. Compounds with mass within 5 parts per million (# Hits at 5 ppm) for the uremic features could be identified within the Human Metabolome Database (www.hmdb.ca) for only a minority of the features characterized as uremic.

Exact Mass (Neutral)	Detected in Polarity	Retention Time (min)	HD/NL Peak Area Plasma UF	HD/NL Peak Area Plasma	# Hits at 5 ppm
**Features characterized as uremic**					
217.98858	NEG	4.42	3058	1766	3
265.09528	NEG	6.21	1584	*	2
380.04178	NEG	5.77	1289	*	0
243.02048	NEG	4.6	996	357	0

262.01498	NEG	5.09	924	242	5
205.98868	NEG	2.6	904	*	3
284.08998	NEG	6.38	734	717	3
280.10542	POS	3.01	733	*	4
275.01058	NEG	4.09	728	258	0
352.07738	NEG	6.38	665	*	1
211.06402	POS	3.40	573	784	1
232.00428	NEG	4.83	546	368	2
189.08202	POS	3.39	538	122	1
189.99378	NEG	2.69	524	535	2
217.03472	POS	3.15	507	*	0
169.03758	NEG	2.83	447	352	2
337.08008	NEG	6.1	441	205	2
208.00428	NEG	3.73	427	369	1
309.08518	NEG	4.72	416	*	3
243.12952	POS	5.23	373	*	0
303.12218	NEG	5.79	368	197	1
246.10002	POS	5.04	361	399	4
133.05252	POS	4.72	360	*	7
129.04232	POS	5.04	357	373	7
310.11468	NEG	5.26	350	*	0
247.08392	POS	5.04	349	451	0
363.18882	POS	8.76	313	*	0
398.01068	NEG	8.25	310	34	0
338.06428	NEG	4.24	298	*	2
264.11032	POS	5.02	294	285	3
183.05292	POS	2.08	285	242	1
375.11602	POS	3.85	285	*	0
242.12688	NEG	5.14	278	244	0
225.06398	NEG	3.91	263	*	2
263.04078	NEG	3.13	263	213	0
128.05822	POS	5.04	255	160	3
380.09868	NEG	4.85	243	*	0
162.99378	NEG	3.55	237	144	1
457.19432	POS	9.63	237	157	0
195.05292	POS	3.14	237	94	11
212.00218	NEG	6.47	236	24	0
320.06732	POS	5.05	236	*	0
332.99188	NEG	3.78	236	*	0
266.11698	NEG	5.01	236	243	1
372.09178	NEG	5.06	233	*	0
254.09058	NEG	3.05	231	*	3
146.06908	NEG	5.04	231	291	5
270.07428	NEG	4.39	229	*	2
330.16848	NEG	11.02	225	77	9
440.16888	NEG	9.6	215	158	0
304.07968	NEG	3.16	209	201	1
149.04742	POS	3.58	203	83	3
360.06298	NEG	3.48	203	*	0

308.07402	POS	5.04	200	441	1
131.03692	POS	3.57	197	*	0
366.02598	NEG	4.13	192	*	0
406.18418	NEG	8.97	191	241	0
299.17292	POS	3.21	187	*	2
348.98458	NEG	6.47	185	31	0
448.00158	NEG	6.46	184	280	0
400.08608	NEG	5.06	183	292	0
301.15202	POS	2.57	182	212	0
182.99898	NEG	4.81	181	125	1
256.14172	POS	6.89	179	78	0
193.98848	NEG	2.21	173	*	0
180.05322	POS	3.96	173	107	3
231.97802	POS	8.24	160	22	0
194.06928	NEG	3.19	157	118	2
259.01518	NEG	3.67	156	*	0
363.02712	POS	5.04	153	217	0
138.03168	NEG	4.45	152	69	9
210.06382	POS	2.51	151	*	2
378.15318	NEG	5.86	150	*	0
212.06868	NEG	7.42	150	108	10
288.98708	NEG	6.12	150	63	0
263.03488	NEG	4.85	144	59	0
280.99708	NEG	6.47	142	30	0
104.02612	POS	4.84	141	72	0
343.08362	POS	5.04	141	259	0
405.04048	NEG	5.04	140	*	0
133.05232	POS	6.46	139	55	7
215.00548	NEG	6.47	139	32	0
279.07788	NEG	4.04	137	*	1
200.01458	NEG	10.06	135	32	0
229.00478	NEG	4.24	134	104	0
455.03828	NEG	5.04	133	*	0
275.04678	NEG	4.93	132	*	0
348.06272	POS	5.04	131	223	0
300.13182	POS	3.85	130	50	0
218.10512	POS	5.03	128	*	3
204.00938	NEG	6.13	127	74	1
340.07308	NEG	4.73	126	*	0
332.09858	NEG	5.04	126	231	0
304.93118	NEG	4.86	123	72	0
385.95998	NEG	8.23	123	15	0
329.18312	POS	5.06	122	142	0
316.17152	POS	3.71	122	*	0
409.03348	NEG	5.04	120	174	0
229.00478	NEG	3.42	119	80	0
362.08848	NEG	5.03	118	*	0
247.97348	NEG	3.54	117	65	0
400.01318	NEG	5.07	117	*	0

188.06858	NEG	3.81	115	121	1
174.07132	POS	4.05	112	109	1
135.06848	NEG	4.85	111	66	11
119.03692	POS	3.18	111	86	1
238.13132	POS	8.50	110	*	1
272.02128	NEG	3.78	110	*	0
394.06928	NEG	5.04	110	177	0
210.10052	POS	2.90	109	54	1
213.00968	NEG	6.47	109	30	1
205.07398	NEG	8.19	108	27	6
153.04242	POS	2.71	107	54	6
215.07968	NEG	2.83	107	67	1
117.05762	POS	4.85	107	61	2
223.02132	POS	4.86	104	59	0
286.15262	POS	4.40	103	50	0
179.05818	NEG	4.85	102	58	5
380.16868	NEG	7.41	100	130	3
161.04742	POS	4.84	100	41	5
347.23012	POS	4.91	99	*	0
376.13728	NEG	4.62	95	57	4
315.16782	POS	3.74	95	104	0
198.08918	NEG	7.78	94	43	6
210.10002	POS	2.46	93	51	1
220.00428	NEG	6.1	93	53	0
206.00508	NEG	6.13	92	66	0
297.98728	NEG	6.47	91	21	0
286.06928	NEG	4.13	90	*	3
364.03638	NEG	5.04	90	100	0
313.98258	NEG	3.44	89	74	0
360.10618	NEG	3.81	89	*	2
308.01392	POS	8.23	88	18	0
499.27692	POS	10.92	88	62	0
337.90888	NEG	8.27	86	20	0
325.01128	NEG	3.14	84	*	0
220.08202	POS	2.61	84	138	0
462.05668	NEG	5.05	84	262	0
234.02028	NEG	6.85	84	49	1
296.99228	NEG	3.43	83	46	0
184.99528	NEG	4.81	82	53	0
189.10048	NEG	3.54	80	*	1
407.04188	NEG	5.04	80	*	0
262.89078	NEG	3.57	79	*	0
366.03618	NEG	5.04	78	*	0
233.08962	POS	2.83	76	55	0
181.06258	NEG	4.85	76	40	0
482.25218	NEG	10.94	75	56	1
304.17112	POS	3.24	74	*	0
201.06342	POS	4.57	73	*	0
264.03598	NEG	4.86	73	49	0

343.19882	POS	6.79	72	83	0
205.98848	NEG	4.34	72	32	3
314.10048	NEG	7	71	*	6
287.03898	NEG	4.86	71	41	0
184.07368	NEG	6.18	71	40	7
272.05172	POS	2.69	69	53	0
172.07378	NEG	5.32	69	43	6
320.05132	POS	2.46	68	96	0
205.00458	NEG	3.05	67	*	0
306.92838	NEG	4.88	67	*	0
289.03468	NEG	4.86	67	59	0
393.16312	POS	4.61	66	*	1
315.03348	NEG	4.87	66	58	0
371.97558	NEG	5.63	65	*	0
369.97938	NEG	5.65	65	45	0
317.18332	POS	2.59	65	*	0
247.04568	NEG	4.86	64	49	0
410.04108	NEG	5.04	64	133	0
333.10392	POS	2.96	63	55	0
317.97238	NEG	8.25	63	19	0
258.03122	POS	4.85	62	49	0
399.91128	NEG	4.85	58	*	0
309.01618	NEG	4.85	58	44	0
319.00122	POS	4.85	57	37	0
304.03662	POS	4.85	56	31	0
304.07742	POS	2.36	56	75	0
213.00978	NEG	9.1	56	21	1
303.16772	POS	3.23	55	60	1
409.94008	NEG	4.87	54	39	0
323.98118	NEG	4.86	54	43	0
204.00958	NEG	4.2	53	52	1
268.95418	NEG	4.87	52	43	0
170.02178	NEG	4.38	52	*	4
329.15898	NEG	4.31	50	60	0
369.98558	NEG	4.86	50	45	0
483.28282	POS	11.98	50	58	0
186.08928	NEG	7.1	50	27	2
276.04172	POS	4.85	50	27	0
270.12178	NEG	2.92	49	41	1
191.05848	NEG	5.87	49	*	3
641.34258	NEG	10.09	48	*	1
349.92888	NEG	4.85	48	36	0
291.00622	POS	4.85	48	34	0
359.05592	POS	5.04	48	93	0
341.99128	NEG	4.86	47	43	0
377.00388	NEG	4.86	47	39	0
256.00188	NEG	8.25	47	14	0
276.95698	NEG	4.87	47	36	0
200.10508	NEG	4.59	46	25	7

359.95758	NEG	4.86	46	51	0
108.05738	NEG	8.25	45	15	5
419.96918	NEG	4.87	45	40	0
190.00998	NEG	8.25	44	15	0
365.01658	NEG	4.88	44	62	0
198.05298	NEG	2.23	44	83	6
210.07548	NEG	4.04	43	28	2
303.95688	NEG	5.63	43	25	0
241.98628	NEG	5.64	43	16	0
331.96288	NEG	4.87	43	38	0
373.11988	NEG	8.51	42	28	0
351.92608	NEG	4.84	42	61	0
331.19862	POS	5.93	41	34	0
296.00818	NEG	8.34	41	14	0
272.99218	NEG	8.28	40	10	0
228.11148	NEG	3.65	40	24	4
278.98328	NEG	4.86	40	32	0
278.95498	NEG	4.87	40	32	0
323.98908	NEG	8.27	39	13	0
188.01448	NEG	8.21	39	15	2
340.16292	POS	4.89	39	21	0
204.00958	NEG	8.57	39	11	1
198.10032	POS	2.58	39	47	1
307.07752	POS	2.61	38	24	0
193.07408	NEG	5.64	38	*	10
319.98648	NEG	4.86	38	30	0
306.04138	NEG	5.42	37	17	2
159.08932	POS	2.66	36	32	14
412.13372	POS	4.38	36	44	1
270.15752	POS	8.65	36	24	0
145.07362	POS	2.34	36	10	12
185.06888	NEG	3.16	36	*	0
503.25742	POS	7.14	35	28	0
126.03142	POS	2.98	35	77	8
303.14292	POS	3.35	35	45	1
158.05788	NEG	3.61	35	31	4
311.12318	NEG	2.98	35	34	2
150.05392	POS	2.36	34	37	9
189.01408	NEG	8.25	34	7	0
174.99838	NEG	5.63	33	15	0
309.97368	NEG	5.65	33	14	0
308.11108	NEG	3.45	33	20	0
331.23532	POS	7.48	33	32	1
175.99418	NEG	5.64	32	15	0
216.11022	POS	2.09	32	33	3
258.97628	NEG	5.66	30	14	0
264.03068	NEG	2.24	30	65	2
143.05802	POS	3.88	30	32	5
173.99868	NEG	5.65	30	14	1

265.06248	NEG	2.92	29	*	0
232.00438	NEG	5.93	29	32	2
284.12628	NEG	9.98	28	66	1
383.10818	NEG	3.03	28	36	1
459.23102	POS	6.65	28	25	0
327.15252	POS	4.73	27	11	0
178.11032	POS	3.99	27	13	4
240.11128	NEG	4	26	*	0
337.13682	POS	4.12	26	24	1
204.00948	NEG	7.78	26	11	1
226.09482	POS	2.81	25	27	1
207.05358	NEG	4.61	24	*	2
226.12118	NEG	11.1	24	14	2
333.94508	NEG	8.26	23	8	0
320.11098	NEG	4.12	23	31	0
204.13628	NEG	6.52	23	20	5
117.07908	NEG	2.95	23	*	7
191.06178	NEG	3.93	22	27	1
371.17852	POS	5.44	22	13	0
154.02678	NEG	8.73	22	7	7
179.08032	POS	2.97	21	24	1
323.98592	POS	8.20	21	4	0
142.06318	NEG	3.51	21	*	5
110.03668	NEG	5.02	20	10	6
247.08752	POS	2.73	19	17	0
174.08928	NEG	2.41	19	29	6
207.08978	NEG	6.99	19	21	5
191.98938	NEG	5.01	19	10	0
189.99368	NEG	5.01	18	9	2
266.06972	POS	2.56	18	18	1
244.14248	NEG	6	18	18	4
282.89548	NEG	4.82	18	10	0
138.03178	NEG	8.73	17	9	9
318.07338	NEG	2.28	17	*	5
216.09988	NEG	6.92	17	20	0
278.67952	POS	3.68	16	20	0
128.04738	NEG	3.69	14	25	8
239.97648	NEG	4.29	14	*	0
280.89798	NEG	4.81	14	10	0
287.09868	NEG	2.77	14	9	0
301.18882	POS	5.31	14	12	0
166.04918	NEG	2.22	13	12	5
309.16742	POS	5.03	12	23	2
244.08772	POS	2.55	12	13	1
256.21832	POS	11.87	11	11	0
160.07348	NEG	4.77	11	7	9
512.29898	NEG	11.86	11	11	0
237.10048	NEG	7.45	11	9	2
162.08938	NEG	2.6	11	10	2

177.04598	NEG	3.42	10	10	4
285.09562	POS	2.56	10	7	1
116.04738	NEG	2.39	9	9	8
265.13112	POS	4.35	9	9	0
128.04748	NEG	2.23	9	24	8
278.04452	POS	3.72	8	10	0
459.26152	POS	13.22	8	5	0
184.14648	NEG	12.17	8	12	32
265.94568	NEG	2.29	8	8	0
263.94788	NEG	2.23	8	8	0
268.13128	NEG	12.79	7	4	2
297.08912	POS	3.41	7	10	1
252.12052	POS	3.76	7	6	1
357.25112	POS	11.21	7	6	0
526.27848	NEG	10.83	6	5	0
250.04578	NEG	3.79	6	6	0
294.12208	NEG	4.26	5	5	7
281.11192	POS	2.32	5	5	5
125.08402	POS	3.31	5	5	12
184.12082	POS	3.31	5	5	0
243.02048	NEG	7.28	*	235	0
209.06918	NEG	6.04	*	213	2
195.05328	NEG	8.89	*	155	11
169.05252	POS	8.21	*	138	0
260.03578	NEG	10.49	*	130	0
484.26878	NEG	10.8	*	128	0
426.15338	NEG	8.62	*	87	1
244.08498	NEG	8.25	*	75	2
201.07872	POS	3.29	*	74	0
443.17842	POS	8.68	*	72	0
307.98658	NEG	4.87	*	70	0
339.98428	NEG	6.11	*	66	0
364.97998	NEG	3.48	*	65	0
234.02028	NEG	3.75	*	63	1
256.97272	POS	6.44	*	62	0
410.95548	NEG	6.47	*	60	0
333.00862	POS	6.45	*	51	0
216.00948	NEG	6.58	*	48	0
342.96768	NEG	6.46	*	47	0
364.08348	NEG	8.74	*	46	0
130.04172	POS	8.20	*	28	0
276.03078	NEG	6.19	*	26	1
230.02528	NEG	10.14	*	19	0
547.28332	POS	7.59	*	17	0
350.94838	NEG	8.25	*	12	0
264.03078	NEG	7.16	*	11	2
108.05768	NEG	6.4	*	*	5
150.03152	POS	3.90	*	*	5
157.01962	POS	2.64	*	*	0

161.04732	POS	6.11	*	*	5
173.99898	NEG	4.4	*	*	1
179.02548	NEG	3.3	*	*	1
183.05292	POS	3.04	*	*	1
192.00948	NEG	3.09	*	*	0
193.07372	POS	5.64	*	*	10
193.07408	NEG	6.29	*	*	10
194.04278	NEG	6.11	*	*	9
195.05338	NEG	5.53	*	*	11
202.04282	POS	4.84	*	*	0
205.07702	POS	4.65	*	*	0
212.06868	NEG	3.46	*	*	10
213.02712	POS	2.93	*	*	0
217.00488	NEG	4.23	*	*	0
218.02498	NEG	5.27	*	*	1
219.98448	NEG	4.42	*	*	0
219.99268	NEG	4.41	*	*	2
222.00068	NEG	6.11	*	*	0
223.04848	NEG	5.54	*	*	3
223.08488	NEG	4.99	*	*	2
229.04128	NEG	5.02	*	*	0
231.00048	NEG	4.24	*	*	0
237.02518	NEG	2.83	*	*	0
237.10058	NEG	6.22	*	*	2
247.99938	NEG	3.72	*	*	1
249.06738	NEG	4.14	*	*	0
249.99538	NEG	4.86	*	*	0
255.09012	POS	2.85	*	*	7
262.09462	POS	5.13	*	*	1
265.99228	NEG	4.03	*	*	0
267.11742	POS	5.03	*	*	0
271.01578	NEG	5.04	*	*	0
272.13768	NEG	3.16	*	*	0
274.02602	POS	3.08	*	*	0
277.09868	NEG	7.67	*	*	0
278.03838	NEG	3.79	*	*	0
282.12188	NEG	7.79	*	*	3
285.97638	NEG	4.41	*	*	0
287.07412	POS	6.21	*	*	0
287.12642	POS	3.09	*	*	0
288.08508	NEG	2.73	*	*	2
292.11628	NEG	3.73	*	*	1
294.11088	NEG	9.13	*	*	1
296.09018	NEG	7.57	*	*	1
298.99598	NEG	2.82	*	*	0
300.08478	NEG	5.9	*	*	2
301.11562	POS	6.38	*	*	0
302.05712	POS	5.03	*	*	0
302.06418	NEG	2.56	*	*	1

302.19192	POS	3.50	*	*	0
305.02098	NEG	4.26	*	*	0
313.98268	NEG	4.26	*	*	0
314.06458	NEG	2.24	*	*	3
318.97638	NEG	4.76	*	*	0
321.93408	NEG	4.85	*	*	0
322.16338	NEG	7.49	*	*	1
323.10078	NEG	5.27	*	*	0
323.93048	NEG	4.85	*	*	0
324.13028	NEG	6.88	*	*	0
325.07922	POS	3.84	*	*	2
325.08008	NEG	4.4	*	*	1
326.10098	NEG	8.1	*	*	7
326.13708	NEG	10.99	*	*	3
327.09578	NEG	5.62	*	*	2
327.10668	NEG	5.05	*	*	0
330.09618	NEG	2.68	*	*	1
337.08048	NEG	5.06	*	*	2
339.09578	NEG	2.75	*	*	2
339.09588	NEG	5.93	*	*	2
344.11128	NEG	3.64	*	*	2
345.17822	POS	3.60	*	*	0
347.94678	NEG	4.4	*	*	0
348.08778	NEG	5.04	*	*	0
348.09378	NEG	2.94	*	*	0
359.12038	NEG	4.87	*	*	2
361.10032	POS	2.87	*	*	0
362.00988	NEG	5.06	*	*	0
368.04112	POS	6.39	*	*	0
371.11018	NEG	5.74	*	*	0
374.08718	NEG	5.07	*	*	0
374.12188	NEG	2.96	*	*	1
379.01432	POS	4.39	*	*	0
384.01518	NEG	6.4	*	*	0
384.04058	NEG	4.81	*	*	0
391.98148	NEG	5.07	*	*	0
393.03978	NEG	5.05	*	*	0
410.00188	NEG	5.04	*	*	0
410.06438	NEG	2.91	*	*	1
414.04818	NEG	6.38	*	*	0
414.15088	NEG	8.76	*	*	1
418.04342	POS	4.87	*	*	1
420.06558	NEG	6.4	*	*	0
423.20982	POS	9.01	*	*	0
429.01288	NEG	6.39	*	*	0
433.08138	NEG	5.74	*	*	0
434.98178	NEG	5.07	*	*	0
442.06928	NEG	4.86	*	*	0
445.01058	NEG	5.08	*	*	0

447.00408	NEG	4.86	*	*	0
448.01258	NEG	4.85	*	*	0
448.08658	NEG	4.86	*	*	0
450.06928	NEG	5.06	*	*	0
451.24132	POS	10.67	*	*	0
458.04178	NEG	4.85	*	*	0
475.04528	NEG	4.87	*	*	0
482.03598	NEG	6.39	*	*	0
493.01018	NEG	4.86	*	*	0
503.03938	NEG	4.85	*	*	0
510.05658	NEG	4.85	*	*	0
550.20448	NEG	5.04	*	*	2
597.10332	POS	4.84	*	*	0
612.17528	NEG	5.04	*	*	0
613.06752	POS	4.86	*	*	0
618.19328	NEG	5.04	*	*	0
627.13978	NEG	5.04	*	*	0
628.14748	NEG	5.06	*	*	0
680.16418	NEG	5.05	*	*	0
682.41832	POS	5.04	*	*	0
314.06428	NEG	4.38	456		3
353.15118	NEG	4.15	236		0
220.05848	NEG	3.53	179		1
283.05518	NEG	3.73	121		0
269.03618	NEG	7.77	40		0
330.07758	NEG	7.52	27		0
184.03738	NEG	6.19	23		3
287.99368	NEG	3.77	18		0
205.07408	NEG	7.3	12		6
126.06818	NEG	3.89	9		12
230.06168	NEG	12.3	6		0
357.16878	NEG	16.69	5		1
567.35628	NEG	15.25	5		0
565.33998	NEG	14.26	5		0
182.05798	NEG	3.68	4		13
139.02698	NEG	8.18	4		4
589.33918	NEG	14.2	4		0
227.02548	NEG	9.58		136	0
476.09308	NEG	8.24		85	0
459.98218	NEG	8.24		65	0
260.89278	NEG	3.56		52	0
468.27288	NEG	12.34		46	3
600.21818	NEG	8.23		41	0
241.98898	NEG	6.83		38	0
120.05768	NEG	10.05		35	8
267.97688	NEG	4.76		34	0
360.08838	NEG	12.04		33	0
256.09528	NEG	9.39		25	0
245.04848	NEG	10.96		22	0

314.04418	NEG	10.97		19	1
246.05658	NEG	10.96		13	0
160.11008	NEG	12.04		12	13
248.05228	NEG	10.97		11	0
386.17708	NEG	11.64		11	0
681.35492	POS	16.09		11	0
227.02558	NEG	11.09		11	0
226.08458	NEG	11.17		10	6
331.03418	NEG	10.97		10	0
408.17562	POS	13.21		9	0
224.01468	NEG	11.2		7	0
625.34788	NEG	11.88		7	1
464.23832	POS	14.70		7	0
397.25162	POS	13.44		7	0
388.28212	POS	13.91		7	0
410.26402	POS	13.89		6	0
374.26652	POS	13.46		6	0
583.34012	POS	14.16		6	0
422.19092	POS	13.68		6	0
388.19258	NEG	10.85		5	0
396.24852	POS	13.45		5	0
403.22022	POS	13.28		5	0
508.23198	NEG	14.04		5	4
382.23262	POS	12.99		5	0
378.20112	POS	14.35		5	1
386.17728	NEG	10.57		5	0
513.27658	NEG	14.44		5	1
436.20672	POS	13.96		5	0
354.20132	POS	12.14		4	0
360.25072	POS	12.99		4	0
511.32722	POS	12.16		4	0
342.24002	POS	12.96		4	1
300.08808	NEG	5.06	*		0
310.06308	NEG	4.58	*		1
316.07968	NEG	3.82	*		0
327.09578	NEG	4.37	*		2
333.10362	POS	3.82	*		0
341.89708	NEG	4.87	*		0
356.11088	NEG	8.38	*		3
398.10768	NEG	6.82	*		0
420.09178	NEG	4.82	*		0
422.08768	NEG	4.85	*		0
424.95308	NEG	5.06	*		0
426.95018	NEG	5.08	*		0
482.98178	NEG	4.85	*		0
542.99258	NEG	4.83	*		0
575.12048	NEG	4.85	*		0
592.19318	NEG	5.05	*		0
643.11048	NEG	4.86	*		0

653.08818	NEG	5.06	*		0
164.98988	NEG	3.55		*	0
187.06282	POS	10.71		*	2
227.02558	NEG	7.57		*	0
229.02128	NEG	9.6		*	0
233.01608	NEG	7.28		*	0
237.10038	NEG	2.38		*	2
245.01608	NEG	4.6		*	0
259.01528	NEG	6.93		*	0
267.97158	NEG	8.26		*	0
274.01518	NEG	8.71		*	2
277.97568	NEG	6.46		*	0
295.01328	NEG	9.58		*	0
298.96938	NEG	3.57		*	0
301.10542	POS	8.25		*	0
311.00798	NEG	4.63		*	0
312.00338	NEG	9.59		*	0
323.10088	NEG	7.56		*	0
327.09472	POS	5.64		*	2
327.99808	NEG	4.62		*	0
378.99548	NEG	4.62		*	0
392.21682	POS	14.87		*	0
407.08368	NEG	2.73		*	0
421.08818	NEG	8.26		*	0
449.11808	NEG	8.26		*	0
449.99758	NEG	6.46		*	0
455.03788	NEG	3.9		*	0
469.05428	NEG	2.7		*	0
524.33392	POS	14.64		*	0
572.18738	NEG	5.63		*	0
**Features not characterized as uremic**					
116.04748	NEG	3.27			8
118.06308	NEG	2.28			14
118.06308	NEG	3.21			14
125.08412	POS	2.72			12
126.10458	NEG	8.63			21
126.10458	NEG	10.31			21
130.06308	NEG	5.8			15
130.06308	NEG	6.45			15
132.07878	NEG	4.62			18
136.05258	NEG	6.04			17
146.05798	NEG	3.28			9
151.06348	NEG	6.42			10
165.07898	NEG	2.21			9
174.08938	NEG	6.7			6
174.12578	NEG	9.57			2
180.06478	NEG	3.67			3
186.05048	NEG	3.23			0
186.08938	NEG	5.97			2

186.08938	NEG	5.39			2
188.10498	NEG	8.6			10
188.10508	NEG	4.84			10
196.11008	NEG	11.78			3
198.05058	NEG	5.8			1
198.05078	NEG	6.45			1
204.09998	NEG	5.44			0
204.10008	NEG	4.26			0
204.10028	NEG	5.65			0
214.03028	NEG	11.37			0
216.09998	NEG	3.94			0
219.11898	NEG	7.43			0
228.04598	NEG	12.05			0
230.11588	NEG	5.15			1
232.00418	NEG	8.19			2
232.13118	NEG	7.42			0
232.13128	NEG	8.7			0
233.06648	NEG	2.21			0
234.11048	NEG	5			0
240.10088	NEG	11.74			4
241.10338	NEG	11.72			0
242.11532	POS	4.54			0
248.02138	NEG	3.22			0
256.09538	NEG	7.75			0
260.13748	NEG	3.63			2
262.14188	NEG	6.22			1
263.14528	NEG	6.19			0
272.05768	NEG	8.59			0
272.07758	NEG	3.24			1
275.18178	NEG	11.7			0
276.15748	NEG	7.79			1
277.96338	NEG	3.66			0
279.96128	NEG	3.61			0
288.05268	NEG	4.28			0
288.19408	NEG	12.22			1
290.17328	NEG	8.95			1
290.95218	NEG	2.19			0
291.17668	NEG	9.01			0
295.03728	NEG	2.21			0
302.20958	NEG	12.68			3
312.23108	NEG	14.13			8
314.13448	NEG	10.34			0
327.13238	NEG	2.38			1
334.04818	NEG	3.24			0
339.90018	NEG	4.86			0
342.16558	NEG	11.72			0
349.01278	NEG	3.24			0
354.15018	NEG	15.95			0
357.10088	NEG	11.73			0

358.16098	NEG	8.96			0
363.02488	NEG	2.25			0
366.14338	NEG	3.28			2
368.16628	NEG	13.06			3
369.06738	NEG	6.4			0
369.10628	NEG	4.52			0
369.19362	POS	14.11			0
370.18208	NEG	13.49			4
380.16578	NEG	16.16			1
382.18218	NEG	16.86			1
383.16888	NEG	16.16			0
385.18528	NEG	16.85			0
392.29338	NEG	13.7			18
395.12098	NEG	2.38			0
398.21328	NEG	13.72			1
408.21508	NEG	10.94			2
409.07218	NEG	11.74			0
409.16828	NEG	16.17			0
411.18428	NEG	16.87			1
414.20828	NEG	12.3			0
416.02048	NEG	2.41			0
416.24268	NEG	12.84			1
419.10088	NEG	11.73			1
425.16368	NEG	16.16			1
426.04978	NEG	2.39			0
427.17918	NEG	16.86			2
429.07508	NEG	2.22			0
436.15418	NEG	13.09			3
442.02068	NEG	5.77			0
444.27288	NEG	13.43			0
445.27638	NEG	13.42			0
450.26228	NEG	13.91			0
453.28588	NEG	14.2			3
460.26848	NEG	12.35			0
474.28358	NEG	13.23			0
477.28588	NEG	13.86			2
479.30158	NEG	14.48			4
488.29918	NEG	14.69			0
488.29938	NEG	13.55			0
501.28738	NEG	13.86			5
502.31478	NEG	13.83			0
513.30718	NEG	13.5			0
525.28638	NEG	13.83			2
530.34578	NEG	14.43			0
539.32278	NEG	13.86			0
541.33928	NEG	14.85			0
543.34458	NEG	14.78			0
557.33408	NEG	12.17			0
581.14018	NEG	4.85			0

584.26468	NEG	17.95			2
587.32338	NEG	13.67			0
590.08778	NEG	4.91			0
591.09268	NEG	4.86			0
591.35438	NEG	14.73			0
609.32708	NEG	14.85			0
611.14478	NEG	5.07			0
613.33918	NEG	14.13			0
614.36798	NEG	11.68			0
626.06318	NEG	4.86			0
633.32638	NEG	14.26			0
657.32688	NEG	14.24			0
674.23478	NEG	17.95			0
677.31438	NEG	14.88			0
701.31448	NEG	14.31			0
745.30258	NEG	14.95			0
758.48398	NEG	15.59			0
