# Supplementary material for: Untargeted mass spectrometry discloses plasma solute levels poorly controlled by hemodialysis
Source: PLoS One. 2017 Nov 16;12(11):e0188315. doi: 10.1371/journal.pone.0188315 (PMC5690664; doi:10.1371/journal.pone.0188315)
Supplement: S3 Table — (PDF) [file pone.0188315.s004.pdf]

S3 Table. Reagent Compounds Compared to Uremic Features

Listed are 63 reagent compounds and whose mass spectrograms were compared with those of uremic features listed in Supplementary Table 2. Fourteen of the reagent compounds were identified as corresponding to uremic features and 49 were not.

Identified	Compound Name	Exact Mass
yes	pyroglutamic acid	129.0423
yes	3-hydroxybenzoate	138.0317
yes	alanyl-glycine	146.0691
yes	isovalerylglycine	159.0893
yes	N-2-furoyl glycine	169.0376
yes	hippuric acid	179.0578
yes	4-pyridoxic acid	183.0529
yes	p-cresol sulfate	188.0145
yes	cinnamoylglycine	205.0741
yes	indoxyl sulfate	213.0097
yes	homovanillic acid sulfate	262.0150
yes	phenylacetylglutamine	264.1103
yes	phenylglucuronide	270.0740
yes	indoxyl beta-D-glucuronide	309.0852
no	p-cresol	108.0574
no	indole	117.0576
no	L-pyroglutamic acid	129.0423
no	indoxyl	133.0525
no	1,5,6,7-tetrahydro-4H-indol-4-one	135.0685
no	acetophenone oxime	135.0685
no	salicylate	138.0317
no	4-hydroxybenzoate	138.0317
no	phenylglyoxylic acid	150.0315
no	2-carboxybenzaldehyde	150.0315
no	4-formylbenzoic acid	150.0315
no	N-phenylglycine	151.0635
no	L-(+)-alpha phenylglycine	151.0635
no	acetaminophen	151.0635
no	3-aminosalicylic acid	153.0424
no	4-aminosalicylic acid	153.0424
no	5-aminosalicylic acid	153.0424
no	4-methyl-2-nitrophenol	153.0424
no	3-amino-4-hydroxybenzoic acid	153.0424
no	salicylhydroxamic acid	153.0424
no	3-hydroxyanthranilic acid	153.0424
no	2-indolecarboxylic acid	161.0474
no	indole-3-carboxylic acid	161.0474
no	quinolinic acid	167.0219

no	gallic acid	170.0218
no	phenolsulfonic acid	173.9987
no	S-carboxymethyl-L-cysteine	179.0255
no	nicotinuric acid	180.0532
no	homovanillic acid	182.0579
no	saccharin	182.9990
no	DL-3,4-dihydroxymandelic acid	184.0374
no	diethyl oxalacetate	188.0686
no	5-hydroxyindole-3-acetic acid	191.0585
no	N-acetylmethionine	191.0613
no	D-galacturonic acid	194.0428
no	D-glucuronic acid	194.0428
no	4-aminohippuric acid	194.0693
no	2-hydroxyhippurate	195.0529
no	vanillylmandelic acid	198.0530
no	syringic acid	198.0530
no	N-acetyl-L-phenylalanine	207.0898
no	4-nitro-D-phenylalanine hydrate	210.0642
no	kinetin	215.0797
no	N-acetylserotonin	218.1051
no	N-acetyl-L tyrosine	223.0849
no	N-acetyl-L-tryptophan	246.1000
no	N-acetyl-D-tryptophan	246.1000
no	Indole-3-acetyl-L-alanine	246.1000
no	L-aspartyl-L-phenylalanine	280.1062
