# Supplementary material for: Untargeted mass spectrometry discloses plasma solute levels poorly controlled by hemodialysis
Source: PLoS One. 2017 Nov 16;12(11):e0188315. doi: 10.1371/journal.pone.0188315 (PMC5690664; doi:10.1371/journal.pone.0188315)
Supplement: S4 Table — (PDF) [file pone.0188315.s005.pdf]

S4 Table. Uremic Features with Mass Values Matching Those of Known Uremic Solutes for Which Reagent Standards Were Not Obtained

Listed are 33 features characterized as uremic in the current study which have mass values matching those of known uremic solutes in the list compiled by Tanaka et al.[1] but for which we did not obtain reagent standards.

Observed Mass (Neutral)	Detected in Polarity	Retention Time (min)	Known Uremic Solute	Mass Error (ppm)
108.05738	NEG	8.25	Benzyl alcohol	1.1
110.03668	NEG	5.02	Pyrocatechol	1.1
116.04738	NEG	2.39	Levulinic acid	-0.7
133.05232	POS	6.46	5-Hydroxyindole	3.6
138.03168	NEG	4.45	3-Hydroxybenzoic acid	0.1
138.03178	NEG	8.73	4-Hydroxybenzoic acid	-0.6
145.07362	POS	2.34	4-Acetamidobutanoic acid	1.9
160.07348	NEG	4.77	Pimelic acid	0.7
166.04918	NEG	2.22	3-Methylxanthine	-0.5
173.99868	NEG	5.65	Phenol sulphate	0.1
174.08928	NEG	2.41	Suberic acid	-0.5
184.12082	POS	3.31	Acisoga	2.1
189.99368	NEG	5.01	Hydroquinone sulfate	-0.4
189.99378	NEG	2.69	Pyrocatechol sulfate	-0.9
193.07372	POS	5.64	Phenylacetylglycine	0.9
195.05328	NEG	8.89	3-Hydroxyhippuric acid	-0.4
195.05338	NEG	5.53	4-Hydroxyhippuric acid	-0.9
200.01458	NEG	10.06	4-Vinylphenol sulfate	-1.4
204.00938	NEG	6.13	3-Methylcatechol sulfate<br>4-Methylcatechol sulfate	-0.9
204.00948	NEG	7.78	2-Methoxyphenol sulfate	-1.4
205.07408	NEG	7.3	Indolelactic acid	-0.9
210.07548	NEG	4.04	1,3,7-Trimethyluric acid	-0.9
216.11022	POS	2.09	N2,N5-diacetylornithine	3.6
228.11148	NEG	3.65	Prolylhydroxyproline	-2.1
281.11192	POS	2.32	1-Methyladenosine	1.7
284.08998	NEG	6.38	p-Cresol glucuronide	-1.3
285.09562	POS	2.56	N4-Acetylcytidine	1.7
297.08912	POS	3.41	5'-Methylthioadenosine	1.6
303.12218	NEG	5.79	Indoleacetyl glutamine	-0.9
311.12318	NEG	2.98	N2,N2-Dimethylguanosine	-0.6
376.13728	NEG	4.62	Riboflavin	2.7
378.20112	POS	14.35	Pentosidine	1.3
412.13372	POS	4.38	N6-Carbamoyl-L-threonyladenosine	1.4

1. Tanaka H, Sirich TL, Plummer NS, Weaver DS, Meyer TW: An Enlarged Profile of Uremic Solutes. *PLoS One,* 10**:** e0135657, 2015
